# Role of the lipid rafts in the life cycle of canine coronavirus

**DOI:** 10.1099/vir.0.070870-0

**Published:** 2015-02

**Authors:** Annamaria Pratelli, Valeriana Colao

**Affiliations:** Department of Veterinary Medicine, University of Bari, Bari, Italy

## Abstract

Coronaviruses are enveloped RNA viruses that have evolved complex relationships with their host cells, and modulate their lipid composition, lipid synthesis and signalling. Lipid rafts, enriched in sphingolipids, cholesterol and associated proteins, are special plasma membrane microdomains involved in several processes in viral infections. The extraction of cholesterol leads to disorganization of lipid microdomains and to dissociation of proteins bound to lipid rafts. Because cholesterol-rich microdomains appear to be a general feature of the entry mechanism of non-eneveloped viruses and of several coronaviruses, the purpose of this study was to analyse the contribution of lipids to the infectivity of canine coronavirus (CCoV). The CCoV life cycle is closely connected to plasma membrane cholesterol, from cell entry to viral particle production. The methyl-β-cyclodextrin (MβCD) was employed to remove cholesterol and to disrupt the lipid rafts. Cholesterol depletion from the cell membrane resulted in a dose-dependent reduction, but not abolishment, of virus infectivity, and at a concentration of 15 mM, the reduction in the infection rate was about 68 %. MβCD treatment was used to verify if cholesterol in the envelope was required for CCoV infection. This resulted in a dose-dependent inhibitory effect, and at a concentration of 9 mM MβCD, infectivity was reduced by about 73 %. Since viral entry would constitute a target for antiviral strategies, inhibitory molecules interacting with viral and/or cell membranes, or interfering with lipid metabolism, may have strong antiviral potential. It will be interesting in the future to analyse the membrane microdomains in the CCoV envelope.

## Introduction

Coronaviruses, a genus in the family *Coronaviridae*, are large, enveloped, positive-sense RNA viruses, 27.6–31 kb in length, responsible for highly prevalent diseases in humans, birds and domestic animals. The one-third 3′ section of the genome contains ORFs encoding the major structural proteins, spike, envelope, membrane, haemoagglutinin-esterase and nucleocapsid proteins. These ORFs are interspersed with several ORFs encoding different non-structural proteins, most of which have unknown functions (Lai & Holmes, 2001; Pratelli, 2006, 2011). In rooted trees, the members of the coronavirus genus consistently form three distinct monophyletic groups, referred to as phylogroups 1, 2 and 3. Canine coronaviruses (CCoVs) are included in phylogroup 1. In view of the recent increase in the number of newly discovered coronaviruses, and ensuing debates and confusion in the literature concerning coronavirus taxonomy, the unofficial, but widely accepted, nomenclature has been proposed to the International Committee on Taxonomy of Viruses (ICTV) Executive Committee, and phylogroups 1–3 were converted into genera designated *Alpha*-, *Beta-* and *Gammacoronavirus*, respectively (Pratelli, 2011). *Deltacoronavirus* is a new genus proposed in July 2013 (ictvonline.org/virusTaxonomy.asp) (Table 1).

**Table 1.** *Coronaviridae* classification and important viruses in the genus *Alphacoronavirus*

Order	Family	Subfamily	Genus	Species
*Nidovirales*	*Coronaviridae*	*Coronavirinae*	*Alphacoronavirus*	*Human coronaviruses 229E, NL63*
*Transmissible gastroenteritis virus* (TGEV)
*Porcine respiratory coronavirus* (PRCoV)
*Porcine epidemic diarrhoea virus* (PEDV)
*Canine coronaviruses* (CCoVs)
*Feline coronaviruses* (FCoVs)
*Miniopterus bat coronaviruses* Bat-CoV-1, HKU8
*Rhinolophus bat coronavirus* Rh-Bat-CoV-HKU2
*Scotophilus bat coronavirus* Sc-Bat-CoV-512
*Betacoronavirus*	
*Deltacoronavirus*	
*Gammacoronavirus*	
*Torovirinae*		
*Arteriviridae*			
*Mesoniviridae*			
*Roniviridae*			

Lipid rafts are special plasma membrane microdomains with an increased structural order, which are designated liquid ordered domains in model membranes. Lipid rafts, enriched in sphingolipids, cholesterol and associated proteins, play a critical role in different biological aspects of the life cycle of several viruses, and are involved in many processes in viral infection. In particular, the tight packaging of the sphingolipids is maintained by the presence of cholesterol, a major constituent of the lipid rafts, and several proteins partition into these membrane domains (Imhoff *et al.*, 2007). Extraction of cholesterol destroys this order, leading both to disorganization of the lipid raft microdomains and to the dissociation of proteins bound to the lipid rafts (Barman & Nayak, 2007).

The role of cholesterol in the entry of non-enveloped viruses was demonstrated for simian virus 40, rotavirus, rhinovirus and enterovirus (Anderson *et al.*, 1996; Suzuki & Suzuki, 2006). Successful entry of enveloped viruses requires binding to specific cellular receptors and fusion of the viral membrane with the cell membrane. Accumulating evidence suggests that enveloped virus entry may require cholesterol in either, or both, of the two membranes involved. Human immunodeficiency virus (HIV) type 1 infection requires cholesterol both in the target cell membrane and in the viral envelope (Guyader *et al.*, 2002; Liao *et al.*, 2001, 2003). Cholesterol in both membranes is also required for bovine herpesvirus 1 infection of Madin Darby Bovine Kidney (MDBK) cells (Zhu *et al.*, 2010). For other viruses in the subfamily *Alphaherpesvirinae* of the family *Herpesviridae*, such as herpes simplex virus 1, varicella-zoster virus and porcine pseudorabies virus, cell membrane cholesterol is required during virus entry (Bender *et al.*, 2003; Hambleton *et al.*, 2007; Desplanques *et al.*, 2008). Other viruses are sensitive to cholesterol depletion from the cell membrane, such as Semliki Forest virus, murine leukaemia virus, Ebola virus and Marburg virus (Ahn *et al.*, 2002; Bavari *et al.*, 2002; Lu *et al.*, 2002; Phalen & Kielian, 1991). For influenza virus and duck hepatitis B virus, the presence of cholesterol in the viral envelope is critical, but it is not essential in the target cell membrane (Sun & Whittaker, 2003; Funk *et al.*, 2008), and recently it has been demonstrated that canine distemper virus also requires cholesterol in the viral envelope (Imhoff *et al.*, 2007). In contrast, in the case of vesicular stomatitis virus, replication is not affected by cholesterol depletion, and numerous strains of the family *Flaviviridae*, i.e. dengue virus and yellow fever virus, enter and infect cells independent of cholesterol (Umashankar *et al.*, 2008).

It is known that coronaviruses differ in their tissue tropism, and different cellular receptors are involved in virus entry. The depletion of cellular and viral cholesterol inhibits entry of several coronaviruses: mouse hepatitis virus (MHV) (Choi *et al.*, 2005), severe acute respiratory syndrome coronavirus (SARS-CoV) (Li *et al.*, 2007), human coronavirus (HCoV)-229E (Nomura *et al.*, 2004), transmissible gastroeneteritis virus (TGEV) (Ren *et al.*, 2008) and avian infectious bronchitis virus (IBV) (Imhoff *et al.*, 2007). In the present study we investigated the role of cholesterol in the viral envelope and the cell membrane in CCoV infection of A72 cells. Methyl-β-cyclodextrin (MβCD), a cholesterol-binding agent, was employed to remove cholesterol and to disrupt the lipid rafts.

## Results

### Infection efficiency after cholesterol depletion from the cell membrane

**Fig. 1.**
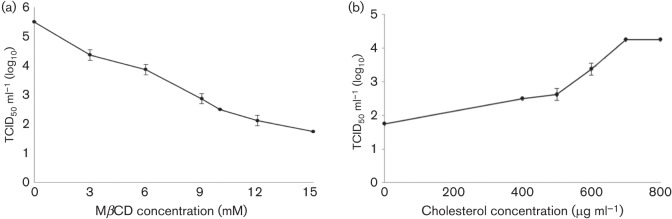
CCoV infection efficiency after cholesterol depletion and replenishment from cellular membrane. (a) MβCD treatment of A72 cells reduced infectivity of CCoV in a dose-dependent manner, and at a concentration of 15 mM, viral titres suffered a reduction of about 68 %. (b) Cholesterol-depleted cells were replenished with exogenous cholesterol and virus production was partially restored. The 100 % infectivity value corresponds to the original titre of the stock virus. Each point indicates the mean value and the error bars represent the standard deviation.

To investigate if cellular cholesterol was essential for CCoV entry into susceptible cells, A72 monolayers were mock pretreated or pretreated with various concentrations of MβCD and subsequently infected with CCoV strain SE/97. Cells were cultured and virus yield was determined with a virus titration assay. MβCD treatment of A72 cells resulted in an abatement of virus production in a dose-dependent manner, suggesting that cell membrane cholesterol is necessary at the entry stage for CCoV infection. At a concentration of 15 mM, the reduction in the infection rate was about 68 % (Fig. 1a).

To confirm that the inhibitory effects for CCoV replication at the entry stage were due to cholesterol depletion, cell membrane cholesterol was replenished with different concentrations of exogenous cholesterol, and the recovery of virus infection was analysed. Cholesterol-depleted cells (pretreated with 15 mM MβCD) were incubated with exogenous cholesterol, infected with CCoV and virus yield was investigated with a virus titration assay. As shown in Fig. 1(b), the inhibitory effect was reversed with cholesterol replenishment and virus production was partially restored to values close to those observed prior to MβCD treatment. At a concentration of 700 µg ml^−1^, infectivity was restored to a mean of 77 % compared to the mock-treated cells.

The concentration of MβCD and cholesterol employed in this study did not cause significant adverse effects on cell viability (data not shown).

### Infection efficiency after cholesterol depletion from the viral membrane

**Fig. 2.**
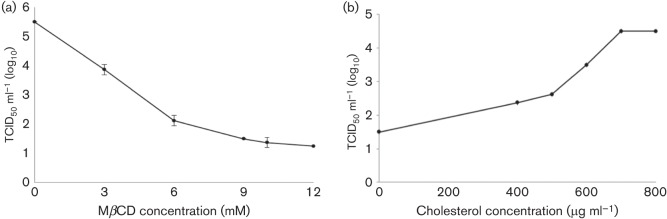
CCoV infection efficiency after cholesterol depletion and replenishment from viral membrane. (a) MβCD treatment of CCoV reduced infectivity in a dose-dependent manner, and at a concentration of 9 mM, viral titres suffered a reduction of about 73 %. (b) Replenishment of cholesterol in the viral membrane resulted in an increase in CCoV infectivity. The 100 % infectivity value corresponds to the original titre of the stock virus. Each point indicates the mean value and the error bars represent the standard deviation.

To analyse whether cholesterol in the viral envelope is required for CCoV entry into susceptible cells, the virus was mock treated or treated with different concentrations of MβCD prior to infection. Cell monolayers were incubated with non-treated and MβCD-treated viral suspensions and virus yield was determined with a virus titration assay. As reported in Fig. 2(a), the exposure of CCoV to MβCD resulted in a dose-dependent inhibitory effect on virus infectivity. In particular, at a concentration of 9 mM MβCD, virus yield was reduced by about 73 %.

To verify whether the effect of cholesterol depletion was reversible, exogenous cholesterol at various concentrations was added to virus suspensions pretreated with 9 mM MβCD. Cholesterol replenishment resulted in an increase of the infectivity of CCoV, and at concentration of 700 µg ml^−1^, infectivity reached about 82 % of the value observed prior to cholesterol depletion (Fig. 2b).

### Cellular and viral cholesterol measurements

**Fig. 3.**
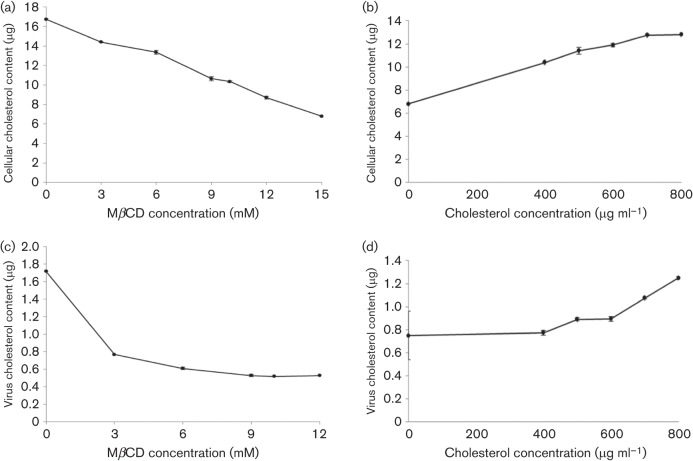
Cholesterol content determination after depletion and replenishment of cholesterol from the cell membrane (a, b) and from the viral membrane (c, d). (a) Cellular cholesterol depletion with various concentrations of MβCD. (b) Recovery of cellular cholesterol after exogenous cholesterol replenishment. (c) Viral cholesterol depletion with various concentrations of MβCD. (d) Recovery of viral cholesterol after exogenous cholesterol replenishment. Each point indicates the mean value and the error bars represent the standard deviation.

A72 cells were treated with various concentrations of MβCD and cellular cholesterol was determined. MβCD treatment resulted in a dose-dependent reduction of the cholesterol content in the lipid raft microdomains of the A72 plasma membrane. In particular, 15 mM of MβCD reduced the amount of cellular cholesterol by about 60 % (Fig. 3a). A72 pretreated with 15 mM of MβCD were analysed after cholesterol replenishment by addition of exogenous cholesterol in increasing amounts. As shown in Fig. 3(b), 700 µg ml^−1^ of exogenous cholesterol restored the cholesterol values of the cell membranes to nearly the values determined prior to MβCD treatment.

Viral cholesterol was also measured. MβCD was used to deplete cholesterol, and increasing drug concentrations resulted in a dose-dependent decrease in cholesterol content in the viral membrane. At a concentration of 9 mM MβCD, viral cholesterol was reduced by about 70 % (Fig. 3c). Cholesterol-depleted virions were replenished with exogenous cholesterol in increasing amounts and virus pellets were used for cholesterol measurements. Exogenous cholesterol (700 µg ml^−1^) restored the cholesterol values of the viral membranes to nearly the values determined prior to MβCD treatment (Fig. 3d).

## Discussion

Viruses are intracellular parasites entirely dependent upon the host cell system for replication and spreading. In the case of enveloped viruses, viral nucleocapsid is surrounded by a lipid membrane derived from the infected cell, where glycoproteins are fixed supporting the functions of entry into target cells and/or fusion between viral and cell membranes. The lipid composition of animal membranes is complex, and three main categories of lipids can be distinguished: glycerophospholipids, sphingolipids and sterols. Sphingolipids are main components of animal cell membranes, and sphingomyelin at the plasma membrane is known to be enriched in lipid microdomains forming the so-called lipid rafts, together with cholesterol (Blaising & Pécheur, 2013). These lipids contribute to viral infection by modulating the properties of viral and/or cell membranes during infection, and can thus play a role through their preferential partitioning into the membrane microdomains. Specifically, viral entry brings together virions and host cells that will interact in a subtly controlled step-by-step process. Each step, therefore, relies on a paired combination of lipids and proteins (Blaising & Pécheur, 2013).

Viruses have evolved complex relationships with their host cells and many viruses modulate lipid composition, lipid synthesis and signalling of their host cell (Blaising & Pécheur, 2013). In particular, lipids are essential for the life cycle of several coronaviruses. The depletion of cellular cholesterol inhibits entry of MHV (Thorp & Gallagher, 2004; Choi *et al.*, 2005), SARS-CoV (Li *et al.*, 2007; Glende *et al.*, 2008), HCoV-229E (Nomura *et al.*, 2004) and IBV (Thorp & Gallagher, 2004; Nomura *et al.*, 2004; Li *et al.*, 2007; Imhoff *et al.*, 2007). Ren *et al.* (2008) showed the importance of cholesterol in both the cell and viral membranes for TGEV infection, and in addition a functional analysis suggested that cholesterol depletion affects a post-adsorption step in the TGEV entry process (Yin *et al.*, 2010). Therefore, the importance of cholesterol-rich microdomains appears to be a general feature of the entry mechanism of different viruses and, as far as coronaviruses are concerned, the purpose of this study was to analyse the contribution of lipids in the infectivity of CCoV, and in particular whether cholesterol was important as a constituent of the virus, the host cells, or both. The CCoV life cycle appears to be closely connected to plasma membrane cholesterol. In the case of TGEV and HCoV-229E, the cholesterol dependence is consistent with the presence of porcine and human aminopeptidase N, respectively (Ren *et al.*, 2008). Conversely, MHV and SARS-CoV use different receptors, MHVR and ACE2, respectively, which are non-raft proteins (Thorp & Gallagher, 2004; Warner *et al.*, 2005).Our analysis did not provide evidence that the activities of the S protein, binding to sialic acids and to aminopeptidase N, were reduced in cholesterol-depleted virions. However, optimal infectivity of CCoV requires cholesterol in the plasma membrane. In particular, cholesterol depletion resulted in a reduction, but not abolishment, of virus infectivity, and virus entry may occur also at lower cholesterol levels, but increased cholesterol makes this process more efficient. At a concentration of 700 µg ml^−1^, infectivity was restored to an average of 77 % compared to the mock-treated cells, confirming that the reduction was due to the cholesterol depletion, and that the inhibitory effect was partially reversible.

Interestingly, our study also demonstrated the role of cholesterol in the viral membrane. This data is particularly important because coronaviruses mature by a budding process in the early compartments of the secretory pathway (Tooze *et al.*, 1984), where the content of cholesterol and sphingolipids is lower than in the plasma membrane (Sevlever *et al.*, 1999). As observed for TGEV by Ren *et al.* (2008), our results confirmed the possibility that lipid microdomains exist in the membrane of CCoV, and the low concentration of cholesterol may explain why the infectivity of CCoV *in vitro* is affected by MβCD concentrations lower than those that affect infectivity of other viruses, like HIV and influenza virus. It will be interesting in future studies to analyse the membrane microdomains in the CCoV envelope.

Lipids and receptors for lipids are therefore key players in the early stages of CCoV infection, i.e. entry and fusion. These stages are amenable to antiviral strategies, and molecules inhibiting CCoV entry and/or fusion could be likely to act on extracellular targets, thereby limiting virus-induced cell damage. By analogy, as observed for hepatitis C virus by Blaising & Pécheur (2013), molecules targeting lipids or their receptors could be considered as CCoV-entry inhibitors, and the virus could be employed in an animal model to test coronaviruses antiviral. Viral entry is a key target for antiviral strategies, and molecules and/or drugs interacting with viral and/or cell membranes, or interfering with the function of lipid metabolism regulators, could be considered potential antivirals, and could constitute potent therapeutics against coronavirus infections combined with existing strategies.

Future work may be aimed at addressing the question of whether cholesterol facilitates coronavirus entry by affecting membrane fluidity, or whether other molecular interactions depend on an increased content of cholesterol.

## Methods

### 

#### Cell and viruses.

The A72 canine fibroma cell line, established from a tumour surgically removed from a female 8-year-old Golden Retriever dog (Binn *et al.*, 1980), was employed. The cells were maintained in Dulbecco’s minimal essential medium (DMEM) supplemented with 5 % FCS, and were passaged twice a week. CCoV strain SE/97 (‘SE’ stands for ‘Seeing Eye dogs’, Pennsylvania isolate) was employed throughout the study. The virus, supplied by Professor L. E. Carmichael (Cornell Vet, Ithaca, NY), was isolated on A72 cells from an adult dog with mild enteritis and recovered within a week. SE/97 was propagated on A72 cells and grown in serum-free medium. The viral titre was determined in 96-well microtitration plates with A72 cells and was expressed as TCID_50_ 50 µl^−1^ calculated using the Reed-Muench formula (Reed & Muench, 1938). CCoV-induced cytopathic effect on infected cells was determined based on the appearance of enlarged, bizarrely shaped cells followed by focal cell detachment. The infectivity titre of the stock virus was 10^5.5^ TCID_50_ 50 µl^−1^.

#### Reagents.

Methyl-β-cyclodextrin (MβCD) (C4555, Sigma-Aldrich) is a strictly surface-acting drug that can selectively and rapidly remove cholesterol from the plasma membrane in preference to other membrane lipids (Barman & Nayak, 2007). This cholesterol depletion reagent has been widely employed in studying the effect of both cholesterol depletion and lipid raft disassembly, and current data indicate that it inhibits entry of several viruses (Choi *et al.*, 2005; Li *et al.*, 2007; Nomura *et al.*, 2004; Imhoff *et al.*, 2007; Ren *et al.*, 2008). To remove the plasma membrane cholesterol, concentrations of 3, 6, 9, 10, 12, 15 mM of MβCD in DMEM were prepared.

Water-soluble cholesterol (C4951, Sigma-Aldrich) was employed to replenish cholesterol after extraction of cellular and viral cholesterol using MβCD.

#### Cholesterol depletion and replenishment from cellular membrane at the virus-entry stage.

To remove cholesterol from cellular membranes, cell monolayers seeded in 24-well plates, containing approximately 300 000 A72 cells per well, were washed three times with DMEM and incubated for 30 min at 37 °C in a CO_2_ incubator with serum-free DMEM in the absence (mock cells) or in the presence (treated cells) of MβCD at concentrations of 3, 6, 9, 10, 12 or 15 mM. To determine if cellular cholesterol depletion at the virus-entry stage affects virus replication, MβCD-treated or mock cells were washed three times with DMEM to remove MβCD and incubated with 100 TCID_50_ 50 µl^−1^ of virus suspension at 37 °C for 1 h. Fresh DMEM was then applied and the cells were incubated for 48 h in a CO_2_ incubator. To investigate the infection efficiency, MβCD-treated or mock cells were frozen and thawed three times, and subjected to virus titration in A72 cells as described above.

For cholesterol replenishment, monolayers of A72 cells in 24-well plates, containing approximately 300 000 A72 cells per well, were mock pretreated or pretreated with 15 mM MβCD for 30 min at 37 °C to remove cellular membrane cholesterol as described above. The concentration of 15 mM was selected because in the cholesterol depletion test, it was the optimal MβCD concentration which did not produce collateral effects for A72 cells.

The cells were then washed three times with DMEM, replenished with different concentrations of water-soluble cholesterol in DMEM ranging from 400 to 800 µg ml^−1^ and incubated for 1 h at 37 °C. Mock cells were replenished with serum-free medium. For cell infection analysis after cellular cholesterol replenishment, the cells were washed three times with DMEM, and viral suspensions containing 100 TCID_50_ 50 µl^−1^ were applied to the cell monolayers. The plates were incubated for 1 h at 37 °C (Zhu *et al.*, 2010), fresh DMEM was applied and the cells were incubated for 48 h in a CO_2_ incubator. To investigate the infection efficiency, treated or mock cells were frozen and thawed three times, and subjected to virus titration in A72 cells as described above.

The reduction and the restoration of viral infectivity were converted to percentages to quantify the reduced and restored amounts, respectively.

All experiments were repeated twice under the same conditions.

#### Cholesterol depletion from viral membranes and effects on virus infectivity.

For viral cholesterol extraction, 1 ml of viral suspensions containing 100 TCID_50_ 50 µl^−1^ were incubated with MβCD at concentrations of 3, 6, 9, 10 or 12 mM for 1 h at 37 °C. To determine if the virus cholesterol was essential for CCoV infectivity, after cholesterol depletion from viral membranes, cell monolayers were washed three times with DMEM and then incubated with MβCD-treated viral suspensions at 37 °C for 1 h. To avoid negative effects of MβCD on A72 cells, inocula were diluted 1 : 3 in DMEM before infection. The controls were mock treated. Finally, treated cells and controls were washed three times with DMEM and incubated for 48 h in a CO_2_ incubator. To analyse the infection efficiency after viral cholesterol depletion, the monolayers were frozen and thawed three times, and subjected to virus titration in A72 cells as described above.

For cholesterol replenishment, 1 ml of CCoV suspensions were mock treated or treated with 9 mM MβCD for 30 min at 37 °C, then replenished with different concentrations of water-soluble cholesterol in DMEM ranging from 400 to 800 µg ml^−1^ and incubated for 1 h at 37 °C. The concentration of 9 mM was selected because in the cholesterol depletion test, it was the optimal MβCD concentration which did not produce collateral effects for CCoV. Mock cells were replenished with serum-free medium. For cell infection analysis after viral cholesterol replenishment, the cells were washed three times with DMEM, and cholesterol-replenished or non-replenished (control) viral suspensions were applied to the cell monolayers and incubated at 37 °C for 48 h (Ren *et al.*, 2008). To investigate the infection efficiency, samples were frozen and thawed three times, and subjected to virus titration in A72 cells as described above.

The reduction and the restoration of viral infectivity were converted to percentages to quantify the reduced and restored amounts, respectively.

All experiments were repeated twice under the same conditions.

#### Cellular and viral cholesterol content measurements.

Cellular and viral cholesterol were measured using an Amplex Red Cholesterol Assay kit (A12216, Invitrogen/Life Technologies) according to the manufacturer’s instructions and according to protocols reported by Ren *et al.* (2008).

To determine cellular cholesterol, confluent monolayers of A72 cells grown in six-well plates were treated with different concentrations of MβCD ranging from 3 to 15 mM. At the same time, monolayers of A72 cells in six-well plates pretreated with 15 mM of MβCD were replenished with various concentrations of exogenous cholesterol ranging from 400 to 800 µg ml^−1^. All the monolayers were then washed three times with DMEM, trypsinized with EDTA, centrifuged at 800 at 4 °C for 5 min to remove cellular debris and the pellets were suspended in PBS. The cellular cholesterol concentration was determined in triplicates with an Amplex Red Cholesterol Assay kit. Non-treated A72 cells were used as a control.

To determine viral cholesterol, 1 ml of two different viral suspensions (10^5.5^ TCID_50_ μl^−1^ each) were treated in parallel with MβCD for cholesterol depletion, specifically one suspension with different concentrations of the drug from 3 to 12 mM and the other with 9 mM. Both suspensions were then treated with exogenous water-soluble cholesterol by applying final concentrations ranging from 400 to 800 µg ml^−1^. The suspensions were centrifuged at 800 at 4 °C for 5 min to remove cellular debris and then ultracentrifuged at 140 000 r.p.m. for 1 h at 4 °C. The pellets were suspended in PBS and subjected to cholesterol concentration determination in triplicates with an Amplex Red Cholesterol Assay kit. Non-treated virus was employed as a control.

All experiments were repeated twice under the same conditions.

It should be noted that CCoV was grown in serum-free medium, to avoid cholesterol measurements being affected by serum cholesterol.
